# Improving New Doctors’ Confidence of Starting Work Through Simulation-Based Training

**DOI:** 10.7759/cureus.94859

**Published:** 2025-10-18

**Authors:** Janhvi Shah, Niraj C Doshi, Akash Doshi

**Affiliations:** 1 Oral and Maxillofacial Surgery, Barts Health NHS Trust, London, GBR; 2 Medicine, University College London, London, GBR; 3 Medicine, Barts Health NHS Trust, London, GBR

**Keywords:** doctors in training, foundation year 1, medical education, new graduates, simulation

## Abstract

Introduction

The transition from final year medical student to Foundation Year 1 (FY1) doctor is widely recognised as challenging, with many new FY1s reporting a lack of preparedness. This contributes to increased stress, higher rates of burnout and potential patient safety issues. Although a mandatory shadowing period has been introduced, the structure and content vary greatly and survey data demonstrates FY1s still feel unprepared.

Methods

A free one-day simulation-based teaching course was developed and refined over seven years using an action research model. The course aimed to increase confidence prior to starting FY1. Session content was developed and refined over these years and includes commonly encountered clinical and non-clinical skills. Pre- and post-course surveys measured confidence at starting FY1. Demographic and qualitative feedback was also gathered.

Results

Between 2017 and 2024, a total of 1,406 final year medical students attended the course. There were increasing numbers of international medical students (IMGs) who attended. The pre- and post-course average confidence rating was 1.99 and 3.68, respectively, out of a five-point Likert scale (with 1 being not at all confident and 5 being completely confident). Common themes for improvement from qualitative feedback identified changes in course content and structure. Of these changes, transitioning to a simulation-based course in 2021 led to the largest increase in confidence.

Conclusion

This study highlights the effectiveness of a simulation-based teaching course to improve FY1 preparedness. It can be easily replicated and localised on a wider scale for use in hospital inductions for new doctors.

## Introduction

Every year, almost 10,000 medical students graduate in the UK and start as Foundation Year 1 (FY1s) doctors. Although medical school training aims to equip students with the knowledge and skills required for clinical practice, the transition to working as a resident doctor is difficult and stressful, with many students feeling significantly underprepared [1,2]. This may be due to difficulties in medical school to safely teach skills such as managing acutely unwell patients, prioritising tasks and prescribing, which require experiential learning and practical experience in a safe environment where patients cannot be harmed [1]. This not only impacts the wellbeing of resident doctors but also on patient safety; an increase in mortality in the month following the start of FY1 doctors of 4%-12% has been reported [3,4]. Similar concerns have been reported in the USA and other countries [5]. Additionally, international medical graduates (IMGs) are increasingly commencing work in the UK at FY1 level, compounding the lack of familiarity with how the National Health Service (NHS) works further exacerbating these risks.

While efforts to improve FY1 preparedness have been made, such as the compulsory shadowing period introduced in 2013, there remains considerable anxiety amongst final year medical students. As there is no national curriculum for content needing to be covered during this shadowing period, there is great variation between hospitals and trusts. Positive feedback from Bristol NHS Foundation Trust demonstrating a 45% reduction in self-reported critical incidents by FY1s following the introduction of the compulsory shadowing period suggests these interventions may be useful [6]. Furthermore, we can see from General Medical Council (GMC) survey data looking at preparedness starting FY1 that the proportion of foundation doctors who felt adequately prepared for their first post increased greatly from 2012 to 2013 likely due to the introduction of this shadowing period [7].

More recently during the COVID-19 pandemic, interim FY1 (FiY1) was introduced for one year to manage the increasing burden on the workforce. This provided experience in advance to the standard FY1 start in August and more enhanced responsibilities than the traditional induction programme. Data from this showed that FiY1s reported higher self-reported preparedness and reduced anxiety than non-FiY1s [8]. This shows the benefits of more real-life practice and extended shadowing; however, this would be difficult to implement as during this shadowing period, FY1s are paid, making it a costly solution.

In this paper, we describe the development over seven years of an annual one-day teaching course, targeting the most common scenarios faced by FY1 doctors. An evidence-based approach to course development and improvement was needed; therefore, both qualitative and quantitative data was used to evaluate teaching, and action research model was used to refine the model over the years.

## Materials and methods

Development of the programme

The aims when developing this course were to develop a reproducible, cost-effective and easily implementable in-person course that improved the confidence of final years towards starting FY1. This was done by developing stations on the most commonly encountered conditions and presentations for FY1s. The teaching was designed and delivered by volunteering doctors. The content was then verified by the course leads who have training in postgraduate medical education.

When finalising the course structure and content, consideration was given to the GMC higher learning outcomes [9], developed in 2021. These have become the cornerstone of the foundation programme and outcomes that all FY1s have to show evidence of meeting. These include being an accountable, capable and compassionate doctor, a valuable member of the healthcare workforce and a professional responsible for their own practice and portfolio development. An action research methodology was used to develop the course over multiple years. A cyclical process of planning, acting, observing and reflecting was used to enact change.

The courses were held at different hospitals, in their outpatient department. Each day was divided into a circuit of stations, in which students participated in small groups covering several topics (Table 1). The number of participants per station varied from eight to 10 depending on number of stations and students who attended. The number and duration of stations were modified over the years following guidance from post-course delegate feedback and ongoing research. Through the years, the number of days and locations increased.

**Table 1:** Course stations every year This table provides an overview of the topics covered in each station between 2017 and 2024. It shows the changes in stations and topics over the years following feedback. Some topics stayed consistent such as certain emergencies and DNACPR. An ultrasound cannulation station was added in the later years, and the curriculum adapted to a simulation-based model. These changes were made following a thematic analysis on the qualitative feedback obtained from participants. DNACPR: do not attempt cardiopulmonary resuscitation, ECG: electrocardiogram, GI: gastrointestinal, TTA: to take away (discharge medications prescribed for patients on leaving hospital), ePortfolio: electronic Portfolio (online record of trainee progress, reflections, assessments and skills).

2017	2018	2019	2021	2022	2023	2024
Acute kidney injury and prescribing fluids	Fluids (managing shock)	Hypotensive patient	Sepsis and DNACPR	Sepsis and DNACPR	Sepsis and DNACPR	Sepsis and DNACPR
High/low heart rate and electrolytes	Heart and blood (ECGs and anticoagulation)	Heart and blood (ECGs and anticoagulation)	Upper GI bleed (discharges, TTAs and referrals)	Upper GI bleed (discharges, TTAs and referrals)	Upper GI bleed (discharges, TTAs and referrals)	Upper GI bleed (discharges, TTAs and referrals)
Diabetic emergencies and prescribing	Sweet and salty (diabetic emergencies, electrolyte imbalances)	Sweet and salty (diabetic emergencies, electrolyte imbalances)	ePortfolio and practical skills	ePortfolio and practical skills	ePortfolio and practical skills	ePortfolio and practical skills
Chest pain and hypoxia	Unwell patient (hypoxia)	Hypoxic patient	-	-	Ultrasound cannulation (run at some courses only)	Ultrasound cannulation
Surgical emergencies and anticoagulation	Surgical patient (vomiting, nausea, constipation, emergencies)	Surgical patient (vomiting, nausea, constipation, emergencies)	Surgical patient (prescribing, fluid management and referrals)	Surgical patient (prescribing, fluid management and referrals)	Surgical patient (prescribing, fluid management and referrals)	Surgical patient (prescribing, fluid management and referrals)
Falls, loss of consciousness and the hypotensive/septic patient	Elderly patient (falls and delirium)	Elderly patient (falls and delirium)	Falls and delirium	Falls and delirium	Falls and delirium	Falls and delirium
Prioritising jobs, handover, referrals, discharge letters	Getting it done (TTAs, prioritising jobs, handover, referrals)	Getting it done (TTAs, prioritising jobs, handover, referrals)	Prioritising jobs (handover)	Prioritising jobs (handover)	Prioritising jobs (handover)	Prioritising jobs (handover)

From 2021, a standardised brief was given to all the tutors that was designed by those with postgraduate degrees in medical education. An example of this can be seen in Appendix 1.

Promotion and recruitment 

All participants were final year medical students about to start their FY1 year, and all data from these participants was used. Participants were recruited through social media channels and through medical schools using the Free Open Access Medical Education (FOAMed) resource “Mind the Bleep” [10]. The tutors used were current FY1 and FY2 doctors who volunteered their time.

Feedback

The primary outcome measure was overall confidence for starting FY1. The level of confidence was measured on a five-point Likert scale (with 1 being least confident and 5 being most). A pre- and post-course survey was used to measure this outcome. In addition, qualitative data was collected through free-text answers and by asking “what went well” and “what could be improved.” Demographic information was also collected, including which medical school they graduated from and whether they were an international medical graduate. Ethical considerations were made, and consent gained from all participants to evaluate their feedback while ensuring that all data remained anonymous.

Analysis

To evaluate the impact of the course, mean confidence scores from the pre-course and post-course groups were compared using an independent-samples t-test, assuming unequal variances (Welch's t-test) as data was unpaired in the context of anonymity. The threshold for statistical significance was established at p < 0.01. All analyses were performed using IBM SPSS Statistics software (version 28.0; IBM Corp., Armonk, NY, USA).

Qualitative data from free-text responses were analysed using thematic analysis [11]. Initial codes were generated from the responses and grouped into broader themes. These themes were reviewed and refined. The two overarching categories of themes emerged were clinical content participants requested additional teaching and suggestions related to course structure or delivery.

Themes were mapped using a structured prioritisation and implementation framework. Based on these themes, targeted interventions were designed. Each intervention was evaluated according to three criteria which were frequency of the suggestion, feasibility of implementation within the course constraints and perceived impact, as judged by the faculty. Interventions were classified as high, medium or low priority. Recommended actions were taken for all high-priority items. To close the feedback loop, changes that were implemented for the following year were evaluated through participant feedback, with particular focus on whether the interventions were beneficial.

## Results

**Figure 1:**
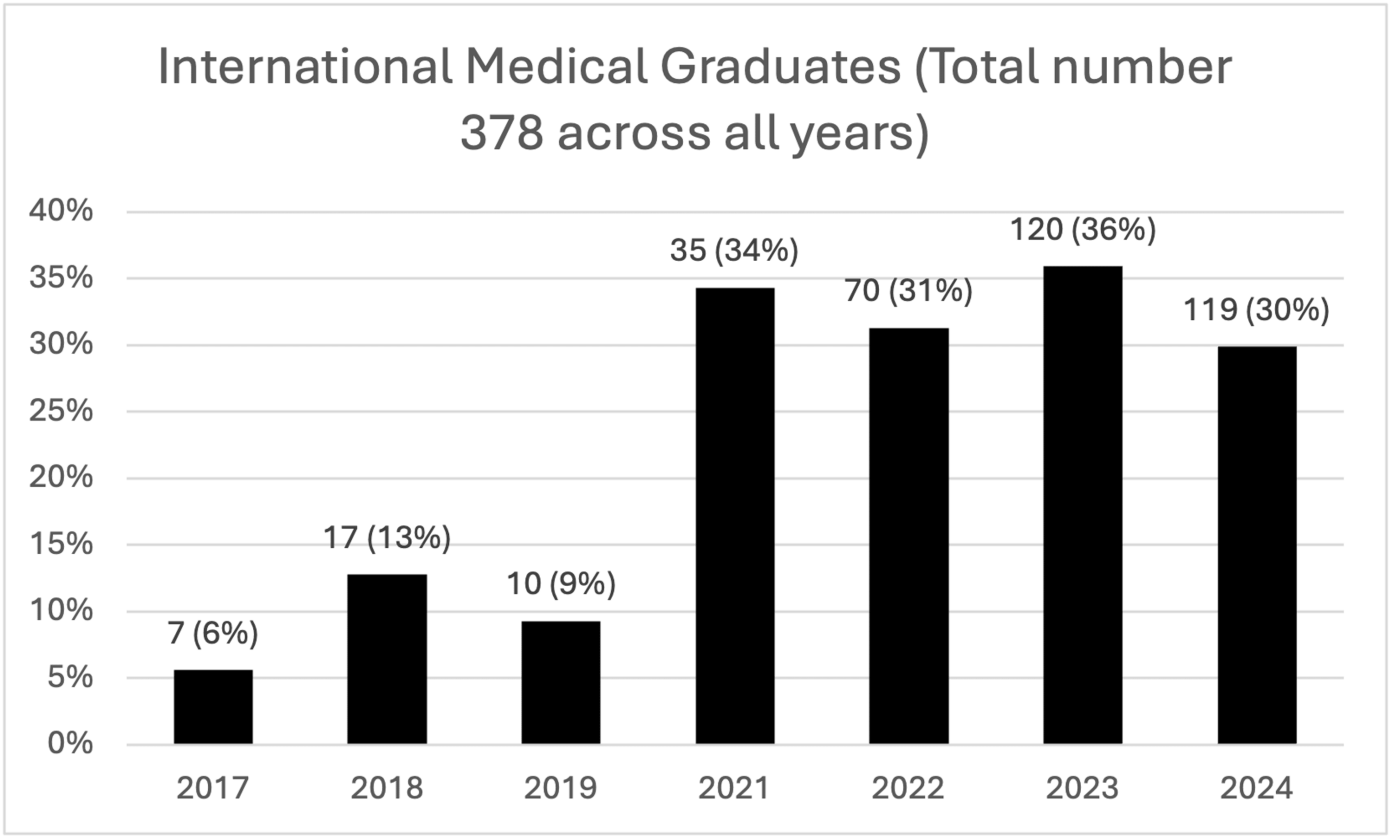
Proportion of international medical graduates This figure illustrates the number of international medical graduates (IMGs) that attended the course each year and as percentage of the total participants. The total number of IMGs over the years was 378. It shows that the proportion of IMGs increased in the later years. The vertical axis represents the percentage, and the horizontal axis indicates the year the course ran. The data labels above each bar display the exact percentage values.

The "Mind the Bleep" course was delivered in person annually between 2017 and 2024, except for the year 2020, to comply with social distancing recommendations for COVID-19 pandemic and due to the impact of the FiY1 programme. A summary of the course is shown in Table 2, and Figure 1 shows the proportion of international medical graduates per year.

**Table 2:** Summary of course by year This table presents a summary of the course by year showing the total number of participants over all the days the course ran, the number of days the course ran that year, the number of locations the course was run at, the number of stations each day, the time per station and the number of tutors per station. It shows the changes that were made each year. It highlights how the course expanded over the years in terms of the number of participants and the number of days/locations. The station time was optimised to 45 minutes, and the number of tutors increased from one to two from 2019 onwards.

Year	Total number of participants	Number of days	Number of locations	Number of stations	Time per station	Number of tutors per station
2017	108	2	1	7	60 minutes	1
2018	133	2	1	7	45 minutes	1
2019	107	2	1	7	30 minutes	2
2021	102	2	1	6	45 minutes	2
2022	225	4	3	6	45 minutes	2
2023	334	7	3	6/7	45 minutes	2
2024	397	8	3	7	45 minutes	2

Quantitative feedback

**Figure 2:**
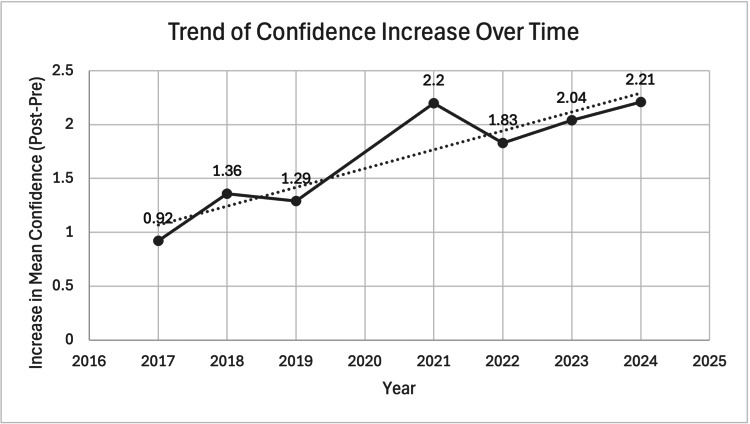
Trend of confidence increase over time This figure illustrates the year-on-year trend in the mean difference between pre- and post-course confidence scores. The graph provides a visual representation of the data. The y-axis represents the mean difference on a five-point Likert scale (1 being not at all confident and 5 being very confident). The x-axis represents the year of the course delivery.

**Figure 3:**
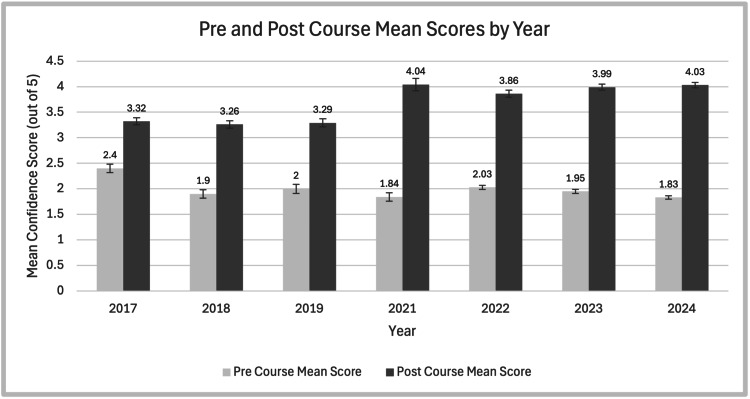
Bar graph showing pre- and post-course confidence with standard error This chart shows the pre- and post-course self-reported confidence scoring with the mean written above each year and with standard error bars. Mean confidence scores from the pre-course and post-course groups were compared using an independent-samples t-test, assuming unequal variances (Welch's t-test). There was a significant difference across all cohorts (p<0.000001). The horizontal axis shows the year, and the vertical axis shows the confidence score on a five-point Likert scale, with 1 being not at all confident and 5 being very confident.

Table 3 shows the mean averages in pre- and post-course confidence (where 1 was not at all confident and 5 completely confident) in starting FY1 over the years. The pre- and post-course average confidence rating was 1.99 and 3.68, respectively. The time trend mean differences can be seen in Figure 2. Figure 3 shows the pre- and post-course confidence each year and the changes seen.

**Table 3:** Pre- and post-course average in confidence levels This table presents the self-reported mean confidence levels and the standard deviation (SD) of participants before and after attending the preparing for Foundation Year 1 (FY1) course across the years from 2017 to 2024. The last column shows the difference in the mean pre- and post-course confidence. The data was collected through a survey, and participants were asked to rate their confidence on a five-point Likert scale where 1 was not confident at all and 5 was very confident. The mean was then calculated from all participants’ data. Across the years, there is a consistent increase in average confidence following the course. Post-course confidence levels remain high across the years, and the largest gain occurred in 2021.

Year	Pre-course, Mean (±SD)	Post-course, Mean (±SD)	Difference in mean pre- and post-course confidence
2017	2.4 (±0.81)	3.32 (±0.58)	0.92
2018	1.9 (±0.93)	3.26 (±0.76)	1.36
2019	2 (±0.89)	3.29 (±0.66)	1.29
2021	1.84 (±0.84)	4.04 (±0.98)	2.20
2022	2.03 (±0.86)	3.86 (±0.99)	1.83
2023	1.95 (±0.94)	3.99 (±1.03)	2.04
2024	1.83 (±0.89)	4.03 (±1.03)	2.21

Qualitative feedback

The high priority interventions identified when asked for improvement to the course have been separated by clinical content (Table 4) and course structure suggestions (Table 5) for improvement. The summary can be found in the tables below with what was implemented the year after.

**Table 4:** Summary of qualitative data gathered showing clinical content participants wanted more teaching This table summarises the high priority suggestions gathered from the qualitative feedback over the years. It then shows what interventions were made for the following year from this feedback. The data shows the changing needs of FY1s and what content was deemed to be more useful by them. Several core content domains were recurring such as prescribing and communication. FY1: Foundation Year 1, IMG: international medical graduate, SBAR: Situation, Background, Assessment and Recommendation.

Year	Key themes from feedback	Interventions implemented the following year
2017	- Emergencies and crash calls - On-call duties - Procedures and practical skills - Answering bleeps and SBAR communication - Specific medical areas e.g. electrolyte imbalances, upper GI bleeds, renal	- Emergency and communication content were included
2018	- Prescribing - Practical skills - On-call scenarios - Practicalities e.g. documentation, handover and prioritising tasks - Acutely unwell patients - Communication with colleagues	- Prescribing practice included - Increased focus on practical aspects on FY1 - Teaching became for scenario based
2019	- Practical skills - Fluid management - Prescribing - Practical aspects e.g. rotas, ePortfolio and applying for leave	- Simulation-based practical teaching was expanded to all stations - Option for practicing practical skills added - Rota/leave advice incorporated
2021	- Prescribing - Rota management and annual leave	- Prescribing practice implemented further
2022	- Referrals - Discharges - Prescribing - Communication	- Communication scenarios (handover/referrals) formalised in station structure
2023	- More time on ultrasound cannulation - More diverse cases - Administrative tasks	- Ultrasound cannulation offered in more locations
2024	- Complex clinical skills e.g. suturing - Managing work schedules, payslips, annual leave - NHS information for IMGs	- IMG-specific resources planned separate to the course

**Table 5:** Summary of qualitative data gathered showing suggestions related to course structure or delivery This table summarises the high priority suggestions gathered from the qualitative feedback over the years. It then shows what interventions were made for the following year from this feedback. This process was used to improve the course for the following year. Early feedback focused on logistics, timing and interactivity. From 2021, feedback included changes to structure and course resources, leading to the introduction of handouts and pre-course packs.

Year	Key themes from feedback	Interventions implemented the following year
2017	- Clarify course location - Shorten registration - Add second tutor per station - Provide learning materials	- Timings altered - Email sent to participants prior to course clarifying details
2018	- Increase number of breaks and shorter stations - Stations to be more interactive and less theoretical - Reduce use of computer presentations	- Introduction of 2 tutors per station - Timings altered
2019	- More interactive stations - Extend duration of stations - Provide resources	- Timings altered
2021	- Increase interactivity - Provide pre-course information	- Pre-course packs were introduced
2022	- More time for reflection and feedback	- Reflection time added with feedback from tutors
2023	- Ensure timing consistency - Summarise station before starting - Handouts - Improve time management	- Handouts and case summaries introduced
2024	- Longer lunch break - Increase variety of teaching methods	- Edits made to brief for tutors

## Discussion

The results demonstrate an improvement in confidence each year, with a significant rise in confidence reported by attendees each year the course has run. The largest increase in reported confidence was when the stations changed from didactic teaching to simulation-based (from 2021). The proportion of international medical graduates (IMGs) has also increased since 2017 and has since remained static at approximately a third of participants since 2021.

The post-course survey following all the courses was used to establish the optimum number of participants per station and the number of tutors. In the first two years of the course, there was only one tutor per station, and multiple comments were made that there was not enough interaction. Therefore, two tutors were used per station, allowing for further interaction and smaller group teaching.

By assessing the feedback, the changes that were suggested included adjustment of the content, teaching methods, timings and focus areas for learning. These were integrated by adjusting the timings, making the teaching simulation-based and adding necessary content while removing less useful content. In the first year, teaching was mainly didactic, focusing predominantly on the clinical aspects of working as a doctor. This was done by the use of presentations in lecture-based stations with the opportunity for questions and discussion after, but minimal interaction and case discussion. The format subsequently evolved to involve more case-based and interactive teaching, with no presentations being used in 2021, and a fully case-based simulation model of teaching was used. Multiple areas of teaching were covered in each station with a combination of clinical, administrative and managerial/leadership skills, whereas prior to 2021, these skills were fully separated by station. This allowed for higher fidelity environments, more applicable to real-life scenarios.

The proportion of IMGs attending the course increased substantially over the seven-year period, with a particularly sharp rise seen in 2021. More doctors joined the workforce from outside the UK than were UK-trained in 2019 [12]. This is likely due to the changes in NHS workforce strategy and law, allowing all medical practitioners to the Shortage Occupation List in the UK, meaning that it is easier for international graduates to enter the NHS workforce and specialty training [13]. This pattern is reflected in the increase in IMGs attending the course.

Literature shows that case or problem-based learning has become more favoured in medical education with more medical schools using this method of teaching as the basis for their courses [14]. It involves a virtual patient that is then discussed in a small group with a tutor directing the conversation [15]. Similarly, the benefits of small group teaching have been well documented. It shows that learning in communities of practice, and integrating individuals within a community that have a similar knowledge base and a common area of interest allows for experience sharing, increased participation and increased participant enthusiasm [16].

The use of simulation in medical education has been growing as it can help educate healthcare professionals in a realistic environment and has been shown to improve patient care and develop non-technical skills [17-19]. The reasons simulation has gained popularity include improving performance in crisis situations, supporting patient safety and that it solves challenges related to medical education [20]. While small group teaching has its advantages and disadvantages, it is ideal for simulation teaching. Potential disadvantages include that participants may not feel comfortable in a group of unfamiliar individuals, causing stress and hypervigilance [21]. However, this can be overcome by tutor experience and ensuring a comfortable environment. It also reflects real-life on-call situations where doctors are often placed in an unfamiliar team.

Looking at the practical implications of this study, we suggest that this course or similar can be rolled out across the UK to allow training for new FY1s. While most simulation-based teaching can be a high-cost endeavour and time-consuming to develop [22], this method allows for easy roll out across NHS trusts as it has already been through rigorous testing and development, without being resource intensive. It can be tailored to include specific computer systems, local processes, prescribing formats and other requirements, as well as be adapted to curriculum needs. The cost of implementing the programme was minimal. The location was in the outpatient department or medical education centre of different hospitals and so was free. The tutors and leaders were all volunteers, and the content was developed for free by the organisers. The tutors and leaders benefited with a letter confirming their role which could be used for their CV. During hospital induction, there is limited time available to cover everything that is needed, and therefore, the use of a one-day course covering most common areas encountered during FY1 allows for more time in induction to cover other aspects. Multimodal teaching like this can simultaneously provide teaching on many aspects of being a doctor.

However, there were some limitations to both the study and the course itself. Firstly “confidence” is self-assessed and therefore does not accurately represent knowledge or clinical abilities. Therefore, using this to assess participants may not be fully in keeping with all the aims of the course. Furthermore, there is possibly an effect of selection bias on these results, given that those who signed up for these courses did so voluntarily and for their own learning. Therefore, they may represent a more anxious or motivated sample of the population. There were also different baseline characteristics of each group year-to-year; however, this reflects the changing demographic of the workforce.

While a standardised brief was given to the tutors, most did not have any formal teaching qualifications. This may have led to inconsistencies in teaching. However, leads were selected with educational experience and qualifications to reduce the risk of this. Prior to the introduction of this brief (prior to 2021), it was found that the feedback varied greatly between stations, largely due to the variety in teaching. To increase consistency further, specific learning objectives were defined for each station and given to the tutors and explained to the participants. There may also be difficulty in recruiting tutors who are volunteering their own time. However, evidence of teaching has become an essential part of nearly all medical portfolios and specialty applications, and an integral part of motivating tutors was aligning their needs to the course. Larger venues and more facilitates may be needed if the course grows further.

In conclusion, this study demonstrates an effective way of increasing the preparedness of final year medical students starting FY1. This model to be refined and adapted continuously to respond to the needs of learners and local education providers. The key outcomes of the action research cycle showed consistent increase in confidence of participants, effective scalability by increasing locations and days and low-cost implementation.

## Conclusions

Due to the positive feedback and advantages of this style of teaching, we recommend that this course be expanded and implemented across the UK as the standard for FY1 induction. Medical schools and hospitals should consider incorporating this programme into FY1 induction alongside the shadowing period. This data has demonstrated the feasibility, efficacy and value of a simulation-based teaching course for new medical graduates before starting FY1. Focused preparation for newly qualified doctors is essential, and many students who attended were incredibly anxious about starting FY1. Ongoing feedback is required to ensure quality control.

## Appendices

Station 1

Summary and Learning Points

In this scenario, a frail patient has been on the ward for several weeks and begins to deteriorate with chest sepsis. They are hypoxic, hypotensive but by applying sepsis 6, there is some improvement in oxygenation and blood pressure. At the end of the station, the patient should deteriorate to the point where a senior attends (e.g. med reg or intensive treatment unit (ITU)) at which point they will recommend that the patient is at their ceiling of care which is ward-based and ITU or CPR is inappropriate. At this point, the station will end.

There is flexibility in this station on what to focus more time on, managing sepsis, family discussions regarding palliation, prescribing and death certification. This learning can occur during simulation or be introduced during the discussion. This will depend on how strong the group is, such that all groups feel adequately challenged. You should spend the bulk of the 45 minutes on discussion rather than simulation as this is where most of the learning comes from. If you try to cram too much into this scenario, attendees will feel rushed and will feel like they’ve missed key learning points.

Specific learning points include (1) Putting out a medical emergency team (MET)/cardiac arrest call and calling for help early. Knowing if you can’t get hold of your seniors, the consultant, Critical Care Outreach Team (CCOT) or ITU is available. (2) Using an ABCDE structure to prioritise appropriate things (e.g. fluids are more important than a catheter if you’re short staffed) and apply the sepsis 6. (3) Using an Airway, Breathing, Circulation, Disability and Exposure (ABCDE) structure to document thoroughly.

After a debrief using the feedback process above, another candidate (one of those sitting down) has to explain to a tutor pretending to be the daughter that their father is for a ward-based ceiling of care and do not attempt cardiopulmonary resuscitation (DNACPR) and may die.

Scenario Outline

Mary Smith, an 86-year-old woman, was admitted four days ago with infective exacerbation of chronic obstructive pulmonary disease (COPD). Now day 4 of doxycycline and was doing better, awaiting physiotherapy and occupational therapy prior to discharge. Today has been lethargic and feeling generally unwell. She has not been engaging in physio, has felt a bit breathless and is coughing discoloured sputum.

The Foundation year 1 doctor (F1) will receive a bleep from a staff nurse on the ward asking to review as they are National Early Warning Score (NEWS) scoring a 9, they are worried [23]. The F1 will have the opportunity to ask for further information and parameters on the phone, but will not be prompted if not asked.

If asked the nurse can give: (1) Previous observations (obs) and latest obs. (2) Appears very breathless and wheezy. (3) Refused dalteparin the last two days. (4) Has been off food and drink all day. (5) Appears slightly confused to time/place.

The patient will appear confused but reveal (1) Felt ok until today, now breathless and wheezy. (2) Coughing up green sputum. (3) Has felt hot and sweaty today.

During the A-E, the F1 should recognise (1) Sepsis: (A) Patent. ​​​​​​​(B) Wheezy bilaterally, crackles right base RR 24 sats 85% on air. ​​​​​​​(C) Heart sounds (HS) 1 + 2, BP 76/35, heart rate (HR) 121, capillary refill time (CRT) 4-5 seconds, ECG sinus tachy. (2) ​​​​​​​The blood pressure will increase to 95/64 after 1.5 L fluid and HR will reduce to 95. ​​​​​​​(D) Glasgow Coma Score (GCS) 14, confused [24]. Not oriented to time/place. Glucose 5.3. ​​​​​​​(E) Abdo soft non-tender (SNT), bowel sounds (BS) present, calves SNT, slightly oedematous ankles, T 39.6. (3) ​​​​​​​Implement sepsis 6, hospital-acquired pneumonia (HAP) and COPD management.​​​​​​​ Implement appropriate investigations such as portable chest X-ray (CXR), arterial gas.

The patient will later deteriorate and the F1 will prescribe preemptive/anticipatory medication and verify the death if time allows.

Baseline: A: Can talk but short sentences. B: Wheezy RR28, sats 85% room air (RA), right basal creps. C: HS normal bp 76/35, hr 121, CRT 4 seconds. D: Slightly confused GCS 14, blood sugar (BM) 6. E: Abdo snt, calves snt. T 39.6.

Deterioration: A: Patent, mumbling. B: RR30, sats 82% 15L non-rebreathe mask (NRM). wheezy. C: BP 95/40, HR 112, CRT 3s. D: GCS 10 E2V3M5 BM 5.0. E: Abdo snt, calves snt, T 37.3.

The F1 should discuss with the registrar for non-invasive ventilation (NIV) who will advise F1 to discuss with patient’s families. Discussion will reveal that the patient has a poor baseline, does not mobilise very well and lives on the ground floor of house as stairs makes her too breathless. She has previously expressed wishes that she "wouldn’t want that tight-fitting mask again, it was awful."

Discussion Points

Discussion points are as follows: (1) Systematic A-E. ​​​​​​​(2) Appropriate investigations. ​​​​​​​(3) Ceilings of care. (4) Ward level, high dependency unit (HDU), ITU. ​​​​​​​(5) Palliative management. ​​​​​​​(6) Death certification.
